# Familial Adenomatous Polyposis Complicated by Acute Myelogenous Leukemia

**DOI:** 10.7759/cureus.8703

**Published:** 2020-06-19

**Authors:** Ala A Alkofahi, Sohaip Kabashneh, Ahmad Alqam

**Affiliations:** 1 Internal Medicine, University of Hawaii, Honolulu, USA; 2 Internal Medicine, Wayne State University/Detroit Medical Center, Detroit, USA; 3 Internal Medicine, King Hussein Cancer Center, Amman, JOR

**Keywords:** familial adenomatous polyposis, acute myeloid leukemia, sporadic

## Abstract

Familial adenomatous polyposis (FAP) is an autosomal dominant disorder characterized by the appearance of multiple colorectal adenomatous polyps and propensity for evolving into adenocarcinoma, typically in early adulthood. We present a case of a 38-year-old man with a one-year history of bloody stool, found to have innumerable polyps throughout the colon and therefore diagnosed with FAP. Completed blood count with differential revealed blasts, a finding confirmed via a peripheral blood smear; a follow-up bone marrow biopsy also showed blasts and he was diagnosed with acute myeloid leukemia (AML). To our knowledge, FAP and AML occurring in the same patient were reported only once in the literature. In our patient and the previously reported case, it is assumed that FAP occurred first and AML developed later; no family history of FAP was noted in either patient. This case raises the question of whether a sporadic FAP is more likely to lead to AML compared to the classic FAP.

## Introduction

Familial adenomatous polyposis (FAP) is an autosomal dominant disorder caused by germline mutations in the tumor suppressor gene, adenomatous polyposis coli (APC), located on chromosome 5q21-q22 [1]. The disorder is characterized by the appearance of multiple colorectal adenomatous polyps (typically more than 100), which typically develops in the second or third decade of life; the mean age at which polyps start to appear is 16 years [2]. If left untreated, almost all patients with FAP develop colon cancer in early adulthood.

FAP has an estimated prevalence of three cases per 100,000 individuals and accounts for less than 1% of all colorectal cancers in the United States [3]. Additionally, FAP can be associated with extracolonic manifestations, and polyps can occur in the upper gastrointestinal tract leading to adenomas in about half of patients [4-6]. Thyroid disorders have also been reported; most patients develop a nodular thyroid, and as many as 12% develop thyroid cancer [7-9]. Brain tumors develop in 1% to 2% of FAP patients, mainly medulloblastoma (80% of cases) [10]. 

Although FAP is frequently associated with extracolonic disease and many tumors in addition to colorectal cancer, it is rarely associated with leukemia. We present a case of FAP complicated by acute myeloid leukemia (AML) in a 38-year-old man.

## Case presentation

A 38-year-old man with no significant past medical history presented to our hospital with recurrent bloody stools for one year.  He described his stool as fresh blood mixed within the stool and on the toilet paper that occurs intermittently and in clusters.  Each cluster lasting four to seven days, during which he experienced three to five bowel movements per day. However, this episode is prolonged (12 days) of continuous bloody stool prompting him to visit the emergency department. He reported subjective fever four days prior to admission, associated with chills, rigors, and night sweat. He denied a change in weight or appetite, change in stool caliber, tenesmus, or abdominal pain. He also denied bleeding from other sites, change in vision, eye redness, oral or perianal ulcer, back pain, or joint swelling or pain. He never had a colonoscopy in the past and was not taking nonsteroidal anti-inflammatory drugs (NSAIDs), antiplatelets, or anticoagulants.  No family history of cancer or inflammatory bowel disease or recent travel. On examination, the patient was afebrile with a temperature of 37°C (98.0°F), a heart rate of 83 beats per minute, and a blood pressure of 135/81 mmHg; his oxygen saturation was 96% on room air and was not in acute distress. Examination of his mouth, lungs, lymph nodes, and heart was unremarkable. His abdominal examination was remarkable for splenomegaly, without tenderness, and his bowel sounds were normal. The digital rectal exam did not reveal external hemorrhoids, perianal fissure, blood, or rectal mass. 

Biochemical and hematological investigations revealed low hemoglobin of 10.1 gm/dL, normal mean corpuscular volume of 88, low platelet of 38 x 10^3^/µL (reference range 151-424 x 10^3^/µL), a normal leukocyte count of 7.03 x 10^3^/µL (reference range: 3.8-10.8 x 10^3^/µL), but low neutrophils 29% (reference range 34%-72%), and surprisingly lab reported blast of 44% (reference range: 0.0%). Basic metabolic profile and liver function tests were within normal.

**Figure 1:**
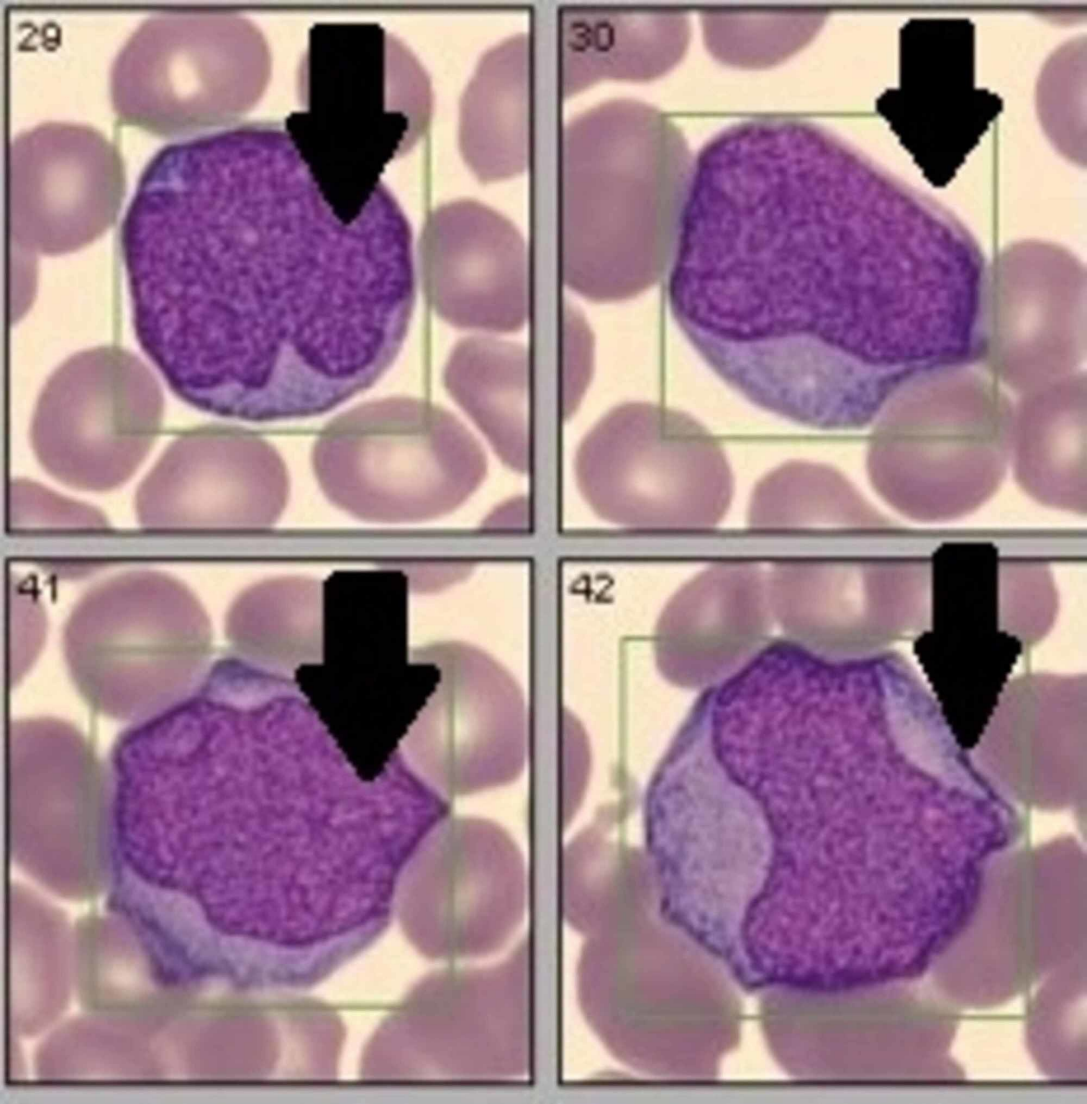
Blood smear shows numerous blasts.

After admission, hematology team was consulted and evaluated the patient`s peripheral blood smear which showed numerous blasts (Figure 1). We then sent for immunophenotyping as per oncology recommendations. The patient continued to experience bloody stool; therefore, he was prepped for a colonoscopy.

**Figure 2:**
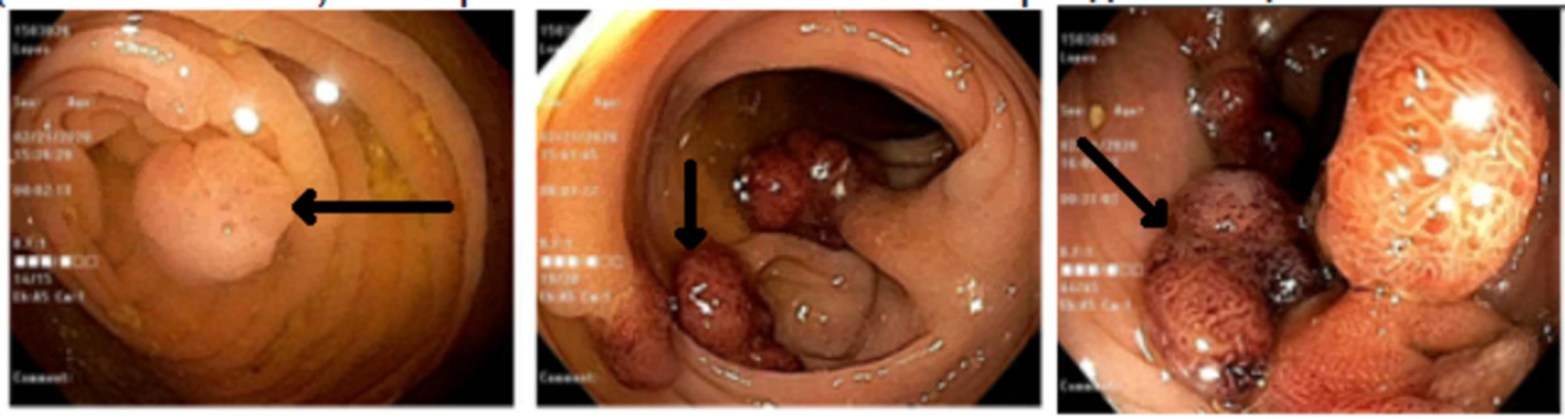
Colonoscopy shows innumerable 2-20 mm polyps throughout the colon.

On the second day of hospitalization, colonoscopy was performed and showed innumerable 2-20 mm polyps throughout the colon, which is consistent with FAP (Figure 2). Later into the hospital course, the results of immunophenotyping came positive for CD33, a finding consistent with AML. Bone marrow biopsy was performed, which showed more than 20% of blasts, and cytogenetics revealed FLT3-ITD and t(6;9) mutations, findings that are consistent with AML.

Chemotherapy (idarubicin and cytarabine) was started later in the hospital course, which he tolerated well. He was discharged, and arrangements were made for him to have a colectomy after managing his AML.

## Discussion

We report a patient diagnosed with FAP and AML simultaneously. To our knowledge, this was reported only once in the literature by Greenberg et al. about 40 years ago, who described two sibships who had this combination [11]. One of them developed multiple adenomatous polyps with adenocarcinoma of the colon and subsequently underwent colectomy at age 35 years. Acute myeloblastic leukemia was discovered at age 45 years. The second brother had multiple colonic polyps and carcinoma of the rectum before the age of 28 years. At age 34 years, he was discovered to have acute myeloblastic leukemia [11]. Our patient had a bloody stool for more than a year prior to his presentation; therefore, it is reasonable to assume he developed FAP first and developed AML afterward because AML is an acute and aggressive disease and patients would not remain asymptomatic for years. In all three patients, FAP developed first and was complicated later with AML.

FAP is an autosomal dominant disorder with nearly complete penetrance of colonic polyposis. However, all three patients had a negative family history suggesting a different mode of inheritance of FAP when it is associated with AML. About 25% of FAP cases arise from de novo APC mutations, and such patients do not have a family history of FAP [12]. It is also reasonable to assume an association between FAP arising from de novo APC mutations and the development of AML.

Alterations of APC gene play a prominent role in the development of colorectal cancer both in the autosomal dominant familial APC syndrome and in sporadic colorectal cancer as it ultimately leads to loss of cellular growth control [13]. In an attempt to understand the association between APC gene and leukemia, Yang et al. investigated the methylation status of the APC gene in adult T-cell leukemia/lymphoma (ATL). The study showed that hypermethylation of the APC gene is involved in the pathogenesis of ATL, which indicates a change in the APC gene can lead to leukemia [14].

## Conclusions

FAP is an autosomal dominant disorder characterized by the appearance of multiple colorectal precancerous polyps. It can be associated with extracolonic manifestations, including thyroid disorders and brain tumors. However, the occurrence of AML was reported only once in the past. In both cases, FAP was sporadic with negative family history and preceded the development of AML. This case highlights the finding that a sporadic FAP seems to increase the risk of AML compared to the classic FAP.
